# Brain Sexual Differentiation and Requirement of SRY: Why or Why Not?

**DOI:** 10.3389/fnins.2017.00632

**Published:** 2017-11-16

**Authors:** Cheryl S. Rosenfeld

**Affiliations:** ^1^Bond Life Sciences Center, University of Missouri, Columbia, MO, United States; ^2^Biomedical Sciences, University of Missouri, Columbia, MO, United States; ^3^Thompson Center for Autism and Neurobehavioral Disorders, University of Missouri, Columbia, MO, United States; ^4^Genetics Area Program, University of Missouri, Columbia, MO, United States

**Keywords:** steroid hormones, sexual dimorphism, neuroscience, mammals, therian, prototherian, Sox9 transcription factor, dopaminergic neurons

## Abstract

Brain sexual differentiation is orchestrated by precise coordination of sex steroid hormones. In some species, programming of select male brain regions is dependent upon aromatization of testosterone to estrogen. In mammals, these hormones surge during the organizational and activational periods that occur during perinatal development and adulthood, respectively. In various fish and reptiles, incubation temperature during a critical embryonic period results in male or female sexual differentiation, but this can be overridden in males by early exposure to estrogenic chemicals. Testes development in mammals requires a Y chromosome and testis determining gene *SRY* (in humans)*/Sry* (all other therian mammals), although there are notable exceptions. Two species of spiny rats: Amami spiny rat (*Tokudaia osimensis*) and Tokunoshima spiny rat (*Tokudaia tokunoshimensis*) and two species of mole voles (*Ellobius lutescens and Ellobius tancrei*), lack a Y chromosome/*Sry* and possess an XO chromosome system in both sexes. Such rodent species, prototherians (monotremes, who also lack *Sry*), and fish and reptile species that demonstrate temperature sex determination (TSD) seemingly call into question the requirement of *Sry* for brain sexual differentiation. This review will consider brain regions expressing *SRY*/*Sry* in humans and rodents, respectively, and potential roles of *SRY*/*Sry* in the brain will be discussed. The evidence from various taxa disputing the requirement of *Sry* for brain sexual differentiation in mammals (therians and prototherians) and certain fish and reptilian species will be examined. A comparative approach to address this question may elucidate other genes, pathways, and epigenetic modifications stimulating brain sexual differentiation in vertebrate species, including humans.

## Introduction

In many sexually reproducing species, the undifferentiated gonad and brain are programmed to be either male or female. As discussed below, in mammalian species that rely on genetic sex determination (GSD), sexual differentiation of the gonad is dependent upon the Y chromosome and the *SRY*/*Sry* gene. However, brain sexual differentiation is generally orchestrated by a surge in gonadal steroid hormones during embryo development followed by a second surge later in adulthood. This paradigm is considered “organization-activational programming” and occurs in a sex-specific manner (Phoenix et al., 1959; Arnold and Breedlove, 1985; Morris et al., 2004). The organizational-activational concept was formulated based initially on findings in guinea pigs (*Cavia porcellus*). Female guinea pigs prenatally exposed to testosterone propionate during a specific developmental window had permanent rewiring of the neural circuitry and as adults, demonstrated male-pattern behaviors (Phoenix et al., 1959). Similar results were then obtained with other mammals (Phoenix et al., 1959; Arnold and Breedlove, 1985; Morris et al., 2004). The early brain programming is termed the organizational period and is characterized by spikes in androgens and/or estrogens such that the brain becomes permanently masculinized (Watson and Adkins-Regan, 1989a,b; Bowers et al., 2010; Konkle and McCarthy, 2011). In the developing mouse brain, sex chromosomes appear to interact with steroid hormones to increase the expression of aromatase expression, which converts androgens into estrogens, and this process appears to be through activation of neural estrogen receptor β (ESR2) (Cisternas et al., 2015, 2017). Masculinization of several brain regions, including the hippocampus, requires aromatization of testosterone to estrogen (Watson and Adkins-Regan, 1989a,b; Bowers et al., 2010; Konkle and McCarthy, 2011). Programming of the male brain though requires both defeminization and masculinization, which generally requires binding of estradiol to its cognate receptors, estrogen receptor-α (ESR1) and ESR2 (Naftolin et al., 1975; Lephart, 1996; McCarthy, 2010). MRI examination of the brain from women with androgen insensitivity syndrome (CAIS), who exhibit a male (46,XY) karyotype but lack functional androgen receptors, reveals that testosterone alone may, however, regulate development of certain brain regions (Savic et al., 2017). These include the somatosensory and visual cortices and their axonal connections to the frontal cortex, along with the connections from the amygdala.

For characteristic sex-dependent behaviors, especially those seen in males, a second spike in steroid hormones at adulthood (“activational period”) is required for full elaboration of sex-specific neurobehavioral pathways (Tsai et al., 2009; Menger et al., 2010; Nugent and McCarthy, 2011; Jasarevic et al., 2012; Matsuda et al., 2012; Chung and Auger, 2013). Steroid hormones at both periods may trigger epigenetic changes in various brain regions that underpin sex differences in brain responses (Tsai et al., 2009; Menger et al., 2010; Nugent and McCarthy, 2011; Jasarevic et al., 2012; Matsuda et al., 2012; Chung and Auger, 2013). However, the question remains as to potential other contributions of sex chromosomes and those genes residing on the X and Y in initiating brain sexual differentiation. One gene residing on the Y chromosome that that has received particular attention is *SRY* (in men)*/Sry* (in other therian mammals). For consistency, the gene annotation form *Sry* will be used throughout the manuscript, unless studies within humans or the human form of SRY are being described.

*Sry* is located on the Y chromosome in therian mammals, which includes placental mammals and marsupials (Delbridge and Graves, 1999; Graves, 2002, 2015; Marshall Graves, 2002; Cortez et al., 2014). SRY functions as a transcriptional activator modulating testis development, and thus it was named the testis determining factor (TDF) in therian mammals (Berta et al., 1990; Sinclair et al., 1990; Foster et al., 1992). The *Sry* gene presumably evolved from a degraded version of the *SOX3* gene on the X chromosome that may itself have originated to guide brain development in both sexes (Graves, 2001). *Sry* is postulated to have evolved before the actual formation of the Y chromosome (Marshall Graves, 2002).

*Sry* encodes a transcriptional factor that includes an HMG box as a DNA domain (Hacker et al., 1995) that activates Sry-related box 9 (*Sox9*) (Sekido and Lovell-Badge, 2008), cerebellin 4 precursor gene (*Cbln4*) (Bradford et al., 2009), *Pod1* (Bhandari et al., 2011), and neurotrophin 3 (*Nt3*) (Clement et al., 2011). Other potential actions of *Sry* include repressing anti-testis forming genes (McElreavey et al., 1993), bending the DNA double helix (Pontiggia et al., 1994), acting as a cofactor within gene silencing complexes (Oh et al., 2005), a splicing factors for pre-mRNA (Ohe et al., 2002); also, the *Sry* transcript might function as a regulator for non-coding RNA (ncRNA) (Hansen et al., 2013). The last three mechanisms imply that *Sry* can stimulate epigenetic responses in the gonad and brain. Other epigenetic mechanisms regulated by *Sry* include DNA methylation, histone protein modifications, and in relation to regulation of ncRNA, *Sry* might act as a potential sponge for microRNAs (miRNAs) (Hansen et al., 2013; Sekido, 2014). This last epigenetic function is determined by which type of *Sry* is transcribed, as detailed below.

The S*ry* gene is intronless, the notable exception being in a single marsupial species, and besides the HMG box region, there is generally poor conservation of the *Sry* genomic sequence across taxa (Sekido, 2010). In mice, two types of *Sry* transcripts can originate depending on whether transcription is signaled through a proximal or distal promoter site (Jeske et al., 1995; Dolci et al., 1997). The first generates a linear *Sry* transcript, which will be translated into a functional protein. The second promoter site gives rise to a transcript that contains an inverted repeat on the 5′ and 3′ end, which results in formation of a stem-loop or circular transcript (Capel et al., 1993). Both transcripts are expressed in the testis and as detailed below, at various points in brain development (Capel et al., 1993; Mayer et al., 2000). Similar to other circular RNAs, the circular form of *Sry* (*circSry*) might act as a “miRNA sponge” that binds to ncRNAs and in the process mitigates their gene silencing ability (Hansen et al., 2013). In relation to the potential role of *Sry* in regulating brain sexual differentiation, *circSry* consists of 16 potential binding sites for miR-148 (Hansen et al., 2013), which is abundant in neuronal and other cell types (Obernosterer et al., 2006; Morton et al., 2008; Eskildsen et al., 2011). In hippocampal neurons, miR-138 inhibits the expression of acyl protein thioesterase 1, an enzyme that mediates depalmitylating α13 subunits of G proteins, which is required for enlargement of dendritic spines (Siegel et al., 2009).

Herein, I will review the evidence to date that *Sry* is involved in brain sexual differentiation in therian species that express this gene. In exploring the potential roles of *Sry* in the brain, we will consider the brain regions where it is expressed, the genes and pathways influenced by *Sry*, and neurobehavioral phenotypes of mice with alterations in *Sry* expression. While *Sry* might be essential for brain sexual differentiation in therian animals, there are mammalian, prototherian, and poikilothermic species that lack *Sry* and/or the Y chromosome. How sexual differentiation, including within the brain, occurs in such species lacking *Sry* will be discussed, along with potential alternative candidates for *Sry*. The primary goal in reviewing the results to date that span several taxa will be to determine whether *Sry* is required for brain sexual differentiation in most therian species and potential other genes that might compensate in *Sry*-deficient species.

## Localization of SRY and potential roles of SRY in brain sexual differentiation

Several studies have examined expression patterns and potential function of *SRY*/*Sry* in the brain in rodent models and humans. Ontogenetic examination of *Sry* expression in the brain of male mice reveals that the circular, presumably non-translated form, is expressed through embryonic days 11–19 (Mayer et al., 2000). After birth, linear, and translatable forms of *Sry* are identified in the diencephalon, midbrain, and cortex. The change from the circular to linear transcripts is presumably driven by a switch in promoter activation, as detailed above. A study with adult mice suggests that *Sry* is expressed in the hypothalamus and midbrain of males but not females (Lahr et al., 1995). Using autopsied samples, SRY protein has been identified in the hypothalamus and frontal and temporal cortex of adult men (Clepet et al., 1993; Mayer et al., 1998).

Another mouse study suggests that *Sry* is expressed in tyrosine hydroxylase (TH)-expressing neurons within the substantia nigra (SN) in adult males (Dewing et al., 2006). Inhibition of *Sry* with antisense oligodeoxynucleotides results in suppression of TH but without reducing neuronal numbers. The reduction in TH in turn leads to motor deficits in treated males. Similarly, SRY protein has been identified in TH-positive neurons within the SN pars compacta (SNc) in human males but not females (Czech et al., 2012). SRY also co-localizes with TH in the ventral tegmental area. When human retinoic-acid treated precursor neuronal NT2 cells differentiate into dopaminergic cells, TH, nuclear receptor related 1 protein (NURR1), dopamine receptor D2 (D2R), and SRY expression are upregulated. Suppression of SRY in human neuroblastoma cell line, M17, results in a depressed expression of TH, DOPA decarboxylase (DDC), dopamine β-hydroxylase (DBH), and monoamine oxidase A (MAO A) expression. These enzymes are primarily involved in dopamine synthesis and metabolism. In contrast, overexpression of SRY elevates the expression of these genes, which is associated with enhanced extracellular dopamine levels. When tested with a luciferase assay, SRY activates an upstream regulatory region within the human TH promoter/nigral enhancer. Dopamine neurons are greater in the SNc in males than females (Murray et al., 2003; Dewing et al., 2006; McArthur et al., 2007). Taken together, the collective results suggest that *Sry*/*SRY* in male mice and humans, respectively, acts as a positive regulator of catecholamine and dopaminergic synthesis and metabolism in the midbrain region. The findings might also help explain why men are more vulnerable to dopamine disorders, namely Parkinson's disease and schizophrenia.

To test further the role of SRY in regulating dopaminergic neurons, human male dopamine M17 cells were treated with a dopaminergic toxin, 6-hydroxydopamine (6-OHDA), which resulted in a significant upregulation of SRY (Czech et al., 2014). Corresponding to this increase, GADD45g, a known SRY regulator, was also elevated. Inhibition of SRY in treated M17 cells increased the levels of reactive oxygen species (ROS), upregulated the pro-apoptotic marker PUMA, and resulted in cell injury. In contrast, overexpression of SRY in 6-OHDA-treated female SH-SY5Y cells was protective and abolished the above cytotoxic effects. However, testing of even greater levels of 6-OHDA or chronic exposure to this toxin resulted in tapered upregulation of SRY, which may be due to gradual apoptosis of dopamine cells.

MAO A is encoded by the X chromosome, and it functions to catalyze the oxidative deamination of monoamine transmitters, including serotonin. SRY can activate the MAO A-promoter and catalytic activities when tested in a human male neuroblastoma BE(2)C cell line (Wu et al., 2009). Co-immunoprecipitation and ChIP assays reveal that SRY and specificity protein 1 (SP1) form a transcriptional complex and synergistically activate MAO A transcription.

*Sry* has been identified in catecholaminergic brain regions of male but not female rats (Milsted et al., 2004). TH is the rate-limiting step of catecholamines. PC12 cells co-transfected with *Sry* and a portion of the TH promoter connected to luciferase exhibit an increase in luciferase reporter activity. Transfection with a shorter construct lacking putative *Sry* sites was also induced by *Sry*. However, transfection with a construct lacking the AP1 site in the TH promoter was not responsive to *Sry*.

## Transgenic mouse models for *Sry* and the Y chromosome

To understand and uncouple the role of *Sry* in the brain from other genes on the Y chromosome, various transgenic mouse models have been created. When human SRY (hSRY) is inserted into single-cell mouse embryos (hSRY^ON^), this early activation of SRY results in impaired brain neurogenesis and several other disorders, including inability of the resulting pups to suckle, development of fatty liver disease, arrested alveologenesis in the lung, sporadic myocardial fibrosis, and thymic hypoplasia (Kido et al., 2017). While the hSRY^ON^ pups are similar in size to wild-type (WT) mice at birth, they undergo retarded postnatal growth and development and die with multi-organ failure before 2 weeks of age. The findings suggest that while SRY might regulate various pathways in the brain and other organs, premature activation of this gene can be deleterious to normal development and physiological function of these organs.

Another approach that has been used to disentangle the role of *Sry* from other Y chromosome genes, has been to delete *Sry* from the Y chromosome and then insert it as a transgene on an autosomal chromosome in both XX and XY chromosome bearing mice (Wagner et al., 2004). XX and XY mice that harbor the *Sry* transgene showed enhanced expression of progesterone receptor immunoreactivity (PRir) in the anteroventral perventricular nucleus (AVPV), the medial preoptic area (MPOA), and the ventromedial nucleus compared to mice lacking the *Sry* transgene. The investigators ascribed the results to the fact that *Sry* transgene upregulated gonadal hormones, and that the results were independent of genetic sex for the brain cells. However, the differences in PRir could also be due to direct effects of *Sry* transgene expression in these brain regions, which was not tested in this study. Co-transfection studies, as detailed above, could be performed to determine if *Sry* directly upregulates the expression of PR, as has been identified with TH.

**Figure 1:**
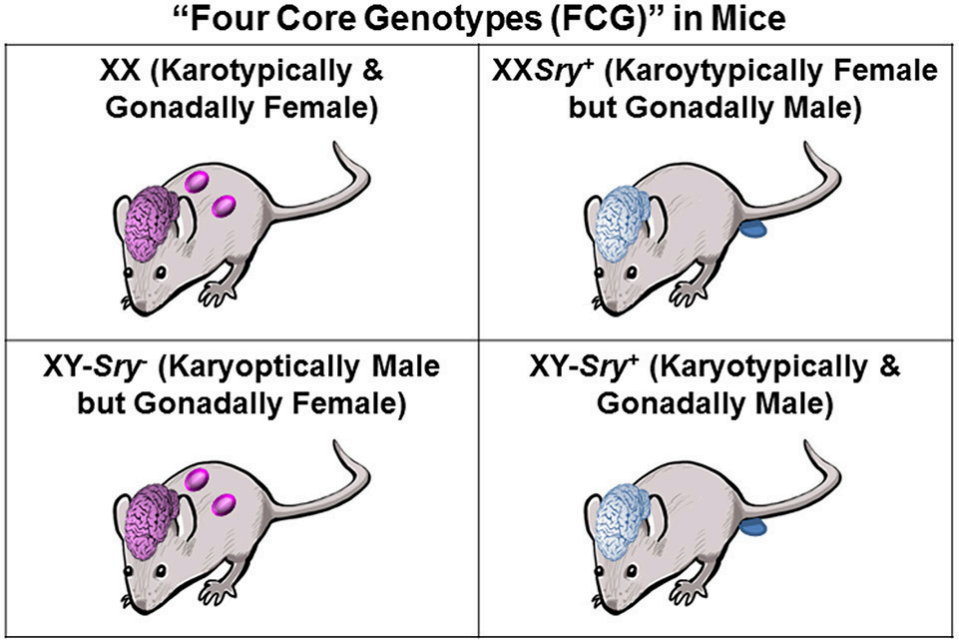
Breeding of XX*Sry*^+^ males with XX females results in offspring of four different genotype/phenotype combinations that in two cases demonstrate mismatches in terms of genetic vs. phenotypic sex of the gonad and likely the brain, as well. The resulting phenotypes could be due to a direct effect of the *Sry* gene or secondary alteration in gonadal sex steroid hormones.

The breeding scheme with this transgenic mouse model above gives rise to the “four-core genotypes—FCG” mouse model (Figure 1; Arnold and Chen, 2009). Offspring from these mated pairs can be one of four different genotypes: XX (karotypically and gonadally female), XX*Sry*^+^ (karoytypically female but gonadally male due to presence of autosomal *Sry* transgene), XY*Sry*^−^ (karyoptically male but gonadally female due to deletion of endogenous *Sry* gene), and XY*Sry*^+^ (karyotypically and gonadally male). Behavioral testing with the FCG model that uncouples the potential effects of *Sry* from other Y chromosome-associated genes and mice gonadectomized at adulthood demonstrates that mice with two XX chromosomes consumed more food during daylight hours and had increased adiposity than XY mice (Chen et al., 2012). However, no *Sry*-related or sex chromosome dependent differences were detected in locomotor or anxiety-related measures (McPhie-Lalmansingh et al., 2008). In this study, however, differences in social behaviors between the FCG were noted. Female XY*Sry*^−^ mice spent more time following and sniffing an intruder compared to XX female mice. XX females were more likely to engage in asocial and digging behaviors compared to XX*Sry*^+^ male mice.

Testing of intact mice with this same model revealed that XX and XY gonadally female (*Sry*^−^) mice were more active, consumed less food per body weight, and when tested in the elevated plus maze (EPM) and zero maze, these groups demonstrated enhanced anxiogenic behaviors compared to XX and XY gonadally male mice (*Sry*^+^) (Kopsida et al., 2013). Another study reported that gonadal males possess greater androgen receptor (*Ar*) expression in the suprachiasmatic nucleus (SCN) and suppressed photic phase-induced behavioral responses compared with gonadal female FCG mice (Kuljis et al., 2013). Adult gonadectomy to remove activational effects of steroid hormones results in XX individuals showing longer activity duration than those with XY chromosomes regardless of gonadal phenotype. Activational effects of gonadal hormones exerted a greater effect in regulating activity levels in gonadal male mice as opposed to gonadal female FCG mice.

The FCG mouse model has also revealed that latency to show aggression and pup retrieval are influenced by gonadal sex and sex chromosome status (Gatewood et al., 2006). Females but not males with XX sex chromosome differ from XY individuals. Vasopressin immunoreactivity in the lateral septum was elevated in gonadal males compared to gonadal females but also differed according to sex chromosome content. The findings suggest genes residing on the sex chromosomes, other than *Sry*, might modulate sex differences in neurobehavioral patterns. When subjected to chronic “stress” conditions, XY mice exhibit reduced expression in the frontal cortex for GABA/serotonin/dopamine-related genes relative to XX mice (Seney et al., 2013). Estradiol treatment of the FCG mice increased growth hormone (*Gh*) expression in the cerebellum and hippocampus independent of sex chromosome complement or gonadal sex (Quinnies et al., 2015). Conversely, in the hypothalamus, females had greater *Gh* expression than males, and XY females showed elevated *Gh* compared to XY males and XX females. In the arcuate nucleus, *Gh* was greater in XX compared to XY mice. Taken together, the findings suggest that sex chromosome complement regulates certain genes within discrete brain regions, but steroid hormones might also play a contributory role.

Transcription activator-like effector nuclease (TALEN) technology is another method that has been used to generate transgenic knockout mice (Sung et al., 2013). *Sry* knockout (KO) mice have been created with TALEN via oocyte injection (Kato et al., 2013). Predictably, *Sry* KO mice possess female external and internal genitalia, low circulating testosterone concentrations, reduced fecundity to complete infertility with decreased number of oocytes but increased luteinized un-ruptured follicles, and feminized neurobehavioral programming. In the AVPV within the MPOA, WT males have greater number of calbindin immuoreactive cells than WT females. *Sry* KO mice exhibit similar number of calbindin positive cells as WT females but lower expression levels relative to that of WT males, particularly in the medial dorsal portion. *Sry* KO mice exhibit female typical reproductive (lordosis) behavior when paired with WT males. Generation of *Sry*-green fluorescent protein (GFP) mice confirmed that *Sry* is expressed in the gonad and brain in day 12 embryos (Wang et al., 2013).

## Rodent species naturally lacking a Y chromosome and SRY

In therian mammals, sex determination is dependent upon the sex chromosome complement and in particular *Sry* on the Y chromosome. However, a handful of notable rodent exceptions have been identified. Two genera that include members lacking the Y chromosome and *Sry* are *Ellobius* and *Tokudaia* (Matthey, 1958; Fredga, 1988; Just et al., 1995; Soullier et al., 1998; Vogel et al., 1998; Sutou et al., 2001). Separate evolutionary events presumably resulted in select species within *Ellobius* and *Tokudaia* shedding the Y chromosome and *Sry* as these genera belong to distinct subfamilies (Arvecolinae and Murinae, respectively). Both of these genera possess an XO system in males and females, and the mechanisms underpinning gonadal and brain sex determination have remained elusive. Detailed below are genomic and gonadal gene expression studies with these two genera. To date, no research group has performed a global assessment of the brain transcriptomic profile in Y chromosome-deficient species within these two genera to determine which genes possibly compensate for the absence of *Sry* expression in the brain of these males.

### Ellobius

The Transcaucasian mole vole (*Ellobius lutescens)* and Zaisan mole vole (*Ellobius tancrei*) lack the Y chromosome and *Sry*, whereas the closely related species, Southern mole vole (*Ellobius fuscocapillus)* and the three other mole voles within this genus have retained the XY system. This loss of the Y chromosome in the two *Ellobius* species likely occurred through two independent evolutionary events that occurred ~0.6 and 2–5 million years ago (MYA) for *E. lutescens* and *Ellobius talpinus*, respectively (Mulugeta et al., 2016). Within the evolutionary conserved region (ECV) of the testis-specific enhancer of SOX9 (TESCO), *E. lutescens, Ellobius trancrei*, and *E. fuscocapillus* display a 14 base pair (bp) deletion removing a highly conserved SOX/TCF site (Bagheri-Fam et al., 2012). This deletion may have resulted in upregulation of *Sox9* in XX gonads and eventual destabilization of the XY/XX determining system in the *Ellobius* genus. *E. lutescens* and *E. trancrei* could have independently evolved *Sry*-independent mechanisms to stabilize sex determination, whereas, *E. fuscocapillus* continued to use *Sry*-dependent mechanisms. *Zfy* is also missing in *E. lutescens* and *E. trancrei* (Vogel et al., 1998). Other genes that have been eliminated as potential replacements for *Sry* in guiding sexual differentiation in *E. lutescens* and *E. trancrei* include nuclear receptor subfamily 5 group A member 1 (*Nr5a1*), dosage-sensitive sex reversal, adrenal hypoplasia critical region, on chromosome X, gene 1 (*Dax1*), steroidogenic factor 1 (*Sf1*), forkhead box protein L2 (*Foxl2*/*Pistr1*), *Sox9, Sox3*, and doublesex and mab-3 related transcription factor 1 (*Dmrt1*) (Baumstark et al., 2001, 2005; Just et al., 2002, 2007).

### Tokudaia

Ryuku (Amami and Tokunoshima) spiny rats (*Tokudaia*) are confined to three islands in the Nansei Shoto archipelago in Japan. The two species though vary in chromosome number with *Tokudaia osmiensis* (residing on the Amami-Oshima Island) having 2n = 25 chromosomes and *Tokudaia tokunoshimensis* (from the Tokunoshima Island) possessing 2n = 45 chromosomes. The Y chromosome and *Sry* are no longer extant in Amami spiny rat (*Tokudaia osimensis*) and Tokunoshima spiny rat (*Tokudaia tokunoshimensis*), and the loss in the two species likely occurred through separate evolutionary events (Soullier et al., 1998; Sutou et al., 2001). The sex-specific region that governs sex determination in XO *Tokudaia* species is likely very minute (Kobayashi et al., 2007). These species possess additional copies of *Cbx2*, which acts upstream of *Sry* (Kuroiwa et al., 2011). Another possible gene that might replace SRY-induced SOX9 expression in the testis of Amami spiny rats is Ets-related protein 71 (*Er71*). *Er71* is also upregulated by SRY in species containing this gene (De Haro and Janknecht, 2005). In conjunction with SF1, SRY binds to TESCO (Sekido and Lovell-Badge, 2008). TESCO activity is lost in this spiny rat, but the activity of the *Sox9* promoter is enhanced by ER71 in this species (Otake and Kuroiwa, 2016). Examination of other Y-linked genes in Amami spiny rats reveal that the original *Rbmy1A1* has also been lost, but *Eif2s3y* and *Kdm5d* have been conserved due to translocation from the Y chromosome to the X chromosome and/or autosomes (Kuroiwa et al., 2010). The other *Rbmy1A1* gene has been reduced to a pseudogene on an autosomal chromosome. Both *Eif2s3y* and *Kdm5d* are expressed in the gonads and brains of both sexes, thus arguing against their possible involvement in sexual differentiation. Testis-specific protein, Y encoded (*Tspy*) and *Zfy* are two other Y-associated genes that translocated to the distal part of the long arm of the X chromosome in the Amami spiny rat (Arakawa et al., 2002), suggesting that these genes are also not the initiators of sexual differentiation in this species.

## Prototherian mammals (monotremes)

Mammals are comprised for two classification groups: therians, which includes placental mammals and marsupials) and prototherians (monotremes or egg-laying mammals). This divergence traces back to 190 million years ago (MYA). Animals within the monotreme order include the duck-billed platypus (*Ornithorhynchus anatinus*) and echidnas (*Tachyglossus aculeatus, Zaglossus* spp., and *Meglibgwilia* spp.). While both monotremes possess five sets of XY chromosomes, they lack *Sry* (Rens et al., 2007; Wallis et al., 2008). Platypuses contain five X and five Y chromosomes that appear to have originated due to translocation of an avian-like sex chromosome (Z) with orthologs of four other chicken chromosomes (Rens et al., 2007; Wallis et al., 2008). The echidna X and Y chromosomes are similar except for the fifth pair. The therian X chromosome resembles platypus autosome 6 and echidna autosome 16 chromosomes (Ferguson-Smith and Rens, 2010). *Sry* is absent in monotremes, and the related gene, *Sox3* (which is present on the X chromosome in therians) resides on chromosome 6 of platypuses and chromosome 16 in echidnas (Wallis et al., 2007b). *Sox9* mediates male sexual differentiation in therians. This gene and related *Sox10* map to platypus autosomal chromosomes 15 and 10, respectively, which argues against both of them being the primary sex determining genes in prototherians (Wallis et al., 2007a). Other genes that have been eliminated as potential sex-determining genes in monotremes are Wilm's tumor 1 (*Wt*1), *Sf1*, LIM Homeobox 1 (*Lhx1*), LIM Homeobox 2 (*Lhx2*), Fibroblast Growth Factor 9 (*Fgf9*), Wnt Family Member 4 (*Wnt4*), R-Spondin 1 (*Rspo1*), and GATA Binding Protein 4 (*Gata4*) (Grafodatskaya et al., 2007). The most likely candidate regulating gonad and potentially brain sexual differentiation in these species is anti-Mullerian hormone (*Amh*) that resides on the shortest and oldest platypus Y5 chromosome (Cortez et al., 2014). Another potential candidate is complex protein gametologs on platypus Y5 (*Crspy*), which is exclusively expressed in the testis and kidneys of males (Tsend-Ayush et al., 2012). There is also a variant on the X1 chromosome (*Crspx*). While the gene(s) stimulating sexual differentiation in monotremes have remained elusive, these species are further evidence that gonad and brain sexual differentiation can occur in the absence of *Sry*.

## Brain sexual differentiation in example fish and reptilian species lacking sex chromosomes and/or *Sry*

This section provides a brief overview of some example fish and reptilian species that lack sex chromosomes and the equivalent to therian *Sry*. Many of these species rely on temperature sex determination (TSD) or other mechanisms to guide sexual differentiation of the gonad and possibly the brain. Several other review articles provide an in-depth discussion of the hormonal control of sexual differentiation in fish (Devlin and Nagahama, 2002; Le Page et al., 2010; Todd et al., 2016; Rosenfeld et al., 2017). The focus herein will be to provide a brief description of these processes in fish and reptilian species and those genes that might guide gonad and brain sexual differentiation in the absence of *Sry*.

### Fish species

Brain sexual differentiation in rodents and other vertebrate species is considered permanent (Arnold and Breedlove, 1985). However, sexualization in fish is strikingly plastic and likely an adaptive mechanism endowed in several fish species to deal with rapid environmental fluctuations occurring throughout an individual's lifespan (Todd et al., 2016). Common reproductive strategies in fish include sequential hermaphrodism where an individual begins life as one sex and then reemerges later on as the opposite sex. Within this category, there are those who exhibit protoandrous (male to female), protogynous (female to male), or serial (bidirectional) sex change. For instance, false clown anemonefish (*Amphiprion ocellaris*) demonstrate protandrous sex changes with a monogamous mating strategy such that a single fish can begin life as a male but then undergo sexual differentiation to become a female, which is likely modulated by transcriptomic changes at the brain and gonad level (Iwata et al., 2012). Sexual plasticity with ability to change gonadal and phenotypic sex is prominent in fish of the teleost lineage. Most teleost fish demonstrate environmental sex determination that is influenced by such factors as water temperature, oxygen levels, pH, fish density, social environment, and age (Nagahama, 2005; Le Page et al., 2010). The primary exception is medaka (*Oryzias latipes*), who demonstrate GSD. In medaka, the DM domain gene on the Y chromomosome/doublesex and mab-3 related transcription factor 1b on the Y chromosome (*dmy*/*dmrt1bY*) and *dmrt1* appear to take the place of therian *Sry* in regulating sexual differentiation (Nanda et al., 2002; Matsuda, 2003; Kikuchi and Hamaguchi, 2013). In this species, *dmy* might stimulate male sexual differentiation by increasing gonadal soma derived growth factor (*gsdf*) and *sox9a2* but suppressing the expression of *rspo1* (Chakraborty et al., 2016). The gene *wt1a* appears to act as an upstream regulator of *dmy*.

Early exposure to exogenous steroid hormones during the critical period of sexual differentiation can alter the sex within select fish (Devlin and Nagahama, 2002). While brain aromatase activity decreases after birth in the mammalian brain, adult teleosts continue to demonstrate high expression of this enzyme due to the upregulation of two aromatase genes (aromatase A—*cyp19a1a* and aromatase B—*cyp19a1b;* Le Page et al., 2010). The latter form is only expressed in radial glial cells (RGC) in adult fish. This persistent expression pattern of neural aromatase in and associated high neurogenic activity suggest that throughout life the brain can be reprogrammed. In gonochoristic (an organism being one of at least two distinct sexes) and hermaphroditic fish species, suppression of *cy19a1a* in the gonad may be required for testicular differentiation (Guiguen et al., 2010). It is uncertain though whether there are SRY-like proteins that regulate the initial sexual differentiation and reprogramming of sex in those that demonstrate sexual plasticity. While fish do not express SRY, several studies have demonstrated that SRY-like proteins are expressed in the gonad and brain.

In Nile tilapia (*Orechromis niloticus*), 27 *sox* genes have been discovered (Wei et al., 2016). It is possible that this gene family has expanded greatly in tilapia and other teleost fishes due to genome duplication. Transcriptome analyses revealed that different sox genes show stage-specific and sex-dependent expression patterns during gonadal development. Six of the group B *sox* genes are exclusively expressed in the adult brain. In medaka, cDNA library screening revealed two forms of *sox9* (*sox9* and *sox9lf*) (Yokoi et al., 2002). The latter included an additional exon in the 5′ upstream region. The *sox9* gene was abundant in the adult ovary, as determined by Northern blot and *in situ* hybridization analyses. In contrast, this gene was only detectable in the testis by qPCR.

Screening of a microarray library and the lamprey (a primitive fishlike and jawless vertebrate) genome revealed genes within the *sox* B, D, E, and F subfamilies (Uy et al., 2012). Within these sub-families, several *sox* genes were expressed during embryogenesis in the neural crest. The rice field eel (*Monopterus albus*) possess at least five *sox* genes (Zhou et al., 2002). The *sox1, sox4*, and *sox14* are intronless in the HMG box region, whereas, *sox9* and *sox17* contain an intron within this conserved region. The rice field eel *sox17* transcript was identified in the gonads of male, female, and intersex individuals (those with an ovotestis), along with the brain and spleen (Wang et al., 2003). Three forms of *sox17* have been identified in European sea bass (*Dicentrarchus labrax*) (Navarro-Martin et al., 2009). One encodes for a typical protein, the second results in a truncated protein, and third shows intron retention. The truncated form of *sox17* was abundantly expressed in the brain and skin, whereas, the normal size transcript increased in the gonad at the onset of sexual differentiation (~150 days of age). From 250 days of age, this *sox17* form was significantly greater in females, and the mRNA expression pattern correlated with gonadal aromatase activity. The promoter of *cyp19a* is hypermethylated in juvenile male sea bass but exposure to an elevated temperature increases *cyp19a* promoter methylation in females, which is associated with decreased expression of this gene (Navarro-Martin et al., 2011). These epigenetic differences are detected in the gonad but not the brain of this fish species. The *sox19* gene is also abundant in the sea bass brain and differentiating ovary but not testis (Navarro-Martin et al., 2012).

The full length form of *sox11a* gene (*Lc-sox11a*) in large yellow croaker (*Larimichthys crocea*) also shows temporal—and sex-dependent expression differences in the gonad and brain (Jiang et al., 2015). In females, *Lc-sox11a* expression was greatest in the ovary followed by the brain, eye, and gill. Conversely, the expression pattern in males was highest in the brain followed by the testis and gill. This transcript was more abundant in the male compared to female brain. With age, *Lc-sox11a* expression increased in the ovary but decreased in the testes. The *sox11a* gene might also be important in neurogenesis in orange-spotted grouper (*Epinephelus coioides*), but it remains to be determine whether there are sex-dependent differences in this species (Zhang et al., 2010).

By screening a testis cDNA library from rainbow trout (*Oncorhynchus mykiss*) followed by Northern blot analyses, *soxlz* was identified, and the protein encoded by this gene bound strongly to an oligonucleotide sequence also recognized by mouse *Sry* and *Sox5* (Takamatsu et al., 1995). Another SRY-related gene, *soxp1*, was identified in the pituitary gland of rainbow trout and showed higher levels of expression in immature fish (Ito et al., 1995).

While *sox* transcripts might stimulate sexual differentiation that results in an increase in testosterone and/or estrogen within the brain or gonad, their expression pattern might also in turn be influenced by endogenous and exogenous steroid hormones. For instance, exposure of adult male zebrafish (*Danio rerio*) for 2 weeks to 25 ng/L ethinyl estradiol (EE2, estrogen present in birth control pills) results in decreased expression of *sox9a* and nuclear receptor subfamily 5 group number 1b (*nr5a2l*) transcripts in the testis that presumably contributes to spawning failure in exposed males (Reyhanian Caspillo et al., 2014). Other expression changes observed in the testis of treated males included an increase in vitellogenin (*vtg*) but a decrease in estrogen receptor-β (*esr2*).

Other sex determining genes characterized in therian mammals have been discovered in different fish species, such as, anti-Müllerian hormone Y (*amhy*) in Patagonian perjerrey (*Odontesthes hatcheri*), *gsdf* in *Oryzias luzonensis* (related to medaka), anti-Müllerian hormone receptor type II (*amhr2)* in tiger pufferfish-fugu (*Takifugu rubripes)*, and sexually dimorphic on the Y chromosome (s*dY*) in rainbow trout (Hattori et al., 2012; Kamiya et al., 2012; Myosho et al., 2012; Yano et al., 2012). Other potential sex determining genes in the brain and gonad of cichlid fish (so-called tribes: Eretmodini, Ectodini, Haplochromini, and Lamprologini) include members of the transforming growth factor-b (*tgfb*) superfamily, Wnt-pathway, *sox*, dm-domain, and high mobility group-box families (Bohne et al., 2014). A follow-up study by this group that tested East African cichlid fish (*Astatotilapia burtoni*) found that the earliest gonadal sexual differentiation markers appeared at 11–12 days post-fry (dpf) and included *wnt4b* and *wt1a* (Heule et al., 2014). Late testis genes in this species were *cyp19a1a, gsdf*, *dmrt1*, and *gata4*, and brain markers were catenin beta 1a *(ctnnb1a)*, catenin beta 1b (*ctnnb1b*), *dax1a, foxl2, foxl3, nanos1a, nanos1b, rspo1*, steroidogenic factor (*sf1*), *sox9a*, and *sox9b*.

Examination of gene expression patterns in the female to male transition in the honeycomb grouper (*Epinephelus merra*), which demonstrates protogynous sex change, revealed that *foxl2* was elevated in the brain, pituitary, ovary, and gill but was downregulated during late transitional stage to male (Alam et al., 2008). In contrast, *dmrt1* expression increased with the initiation of spermatogenesis and complete formation of the testis. This gene might also be important in male sex determination in Chinese tongue sole (*Cynoglossus semilaevis*), a marine fish with ZW sex determination. TALEN injection of *dmrt1* into ZZ mutant fish stimulates ovary-like testis development with disrupted spermatogenesis, an increase female-associated transcripts (*foxl2* and *cyp19a1a*) in the gonad, but reduced expression of male-related genes (*sox9a* and *amh*) (Cui et al., 2017). The *dmrt1* deficient ZZ fish outgrew ZZ male controls.

### Reptilian species

Various patterns of reproductive and sex-determining mechanisms are present within reptiles. Some reptile species exhibit gonochorism, whereas others are unisexual (parthenogenesis). In those with two sexes, sexual differentiation can occur via GSD with male (XX/XY) and female (ZZ/ZW) heterogamety or due to TSD (Ezaz et al., 2009; Wapstra and Warner, 2010). Two hundred out of 1,000 karyotyed reptilian species possess sex chromosomes. Those lacking sex chromosomes are predominantly featured in this section.

In the leopard gecko (*Eublepharis macularius*), incubation temperature of the egg during a critical period of embryonic development modulates gonadal sex and sex-differences in body growth, adult morphology, aggressiveness, reproductive physiology, and neurobehavioral organization and programming (Crews et al., 1998). By altering steroid hormone synthesis, especially for testosterone and estrogen, and increasing expression of either male or female temperature-sensitive transcripts, incubation temperature alone may program early brain development and later behavioral responses. Painted turtles (*Chrysemys picta*) also lack sex chromosomes and demonstrate TSD. When we exposed painted turtles eggs that were incubated at the male producing temperature (MPT) to the endocrine disrupting chemical, bisphenol A (BPA), it resulted in partial gonadal sex reversal to female, feminized their behavioral responses, and altered brain gene expression patterns, especially for transcripts associated with mitochondrial and ribosomal function relative to control males (Jandegian et al., 2015; Manshack et al., 2016, 2017).

There is interest in identifying transcripts in the gonad and/or brain that are altered due to TSD in select reptilian species. Several genes were identified to be elevated in the gonad of American alligators (*Alligator mississippiensis*) after incubation at the MPT (33.5°C), including *wnt11*, histone demethylase *kdm6b*, and transcription factor *cebpa* (Yatsu et al., 2016). The alligator ortholog of the transient receptor potential cation channel subfamily V member 4 (*trpv4*) is another gene associated with the MPT and male sexual differentiation (Yatsu et al., 2015). The genes *sf1* and *wt1* are activated in the gonad of painted turtles during the thermosensitive period and dosage-sensitive sex reversal, and *dax1* may also contribute to male development (Valenzuela et al., 2013).

Australian central bearded dragons (*Pogona vitticeps*) typically demonstrate GSD. However, this can be overridden when eggs are incubated at higher temperatures, which gives rise to sex-reversed female offspring. In females generated through high temperature but not normal chromosomal females, an intron is retained in mature transcripts from two Jumonji family genes, *jarid2* and *jmjd* (Deveson et al., 2017). JARID2 is part of the master chromatin modifier Polycomb Repressive Complex 2, and mammalian *Sry* is regulated by an independent but closely related JUMONJI family member. The same introns are also retained in *jarid2*/*jmjd3* transcripts in embryonic gonads from TSD alligators and turtles. The intron retention in these transcripts might induce epigenetic changes that override chromosomal sex-determining mechanisms, leading to sex reversal at elevated incubation temperatures or a switch from GSD to TSD. These genes might also compensate for the absence of *Sry* and stimulate organizational changes within the brain of these male reptile species.

Investigation of the genome of the lizard *Calotes versicolor*, which demonstrates GSD, with a human *SRY* probe showed hybridization in all males but also in select females (Ganesh et al., 1997). Thus, it is not clear whether *sry* might be involved in sex determination of this reptilian species possessing sex chromosomes. Studies have also been conducted to determine whether *sry*-related genes might compensate for the absence of *sry* in reptiles with TSD. The *sox8* gene is expressed in the gonad of both male and female red-eared slider turtles (*Trachemys scripta*), and thus it is unlikely to be responsible for sex determination in turtles with TSD (Takada et al., 2004). While *dax1* is unlikely to be involved in sexual differentiation in sea turtles (*Lepidochelys olivacea*), upregulation of *dmrt1* and downregulation of *sox9* might exert a role in male and female sexual differentiation, respectively (Torres Maldonado et al., 2002). The fact that *sox9* expression occurs after *amh* suggests that in American alligators (*A. mississippiensis*), it is not the primary initiator of male sex determination (Western et al., 1999a,b). Instead, gonad and brain sex determination in reptilian species with TSD may be due to gene(s) unrelated to *Sry*, such as, those listed previously.

## Discussion

**Figure 2:**
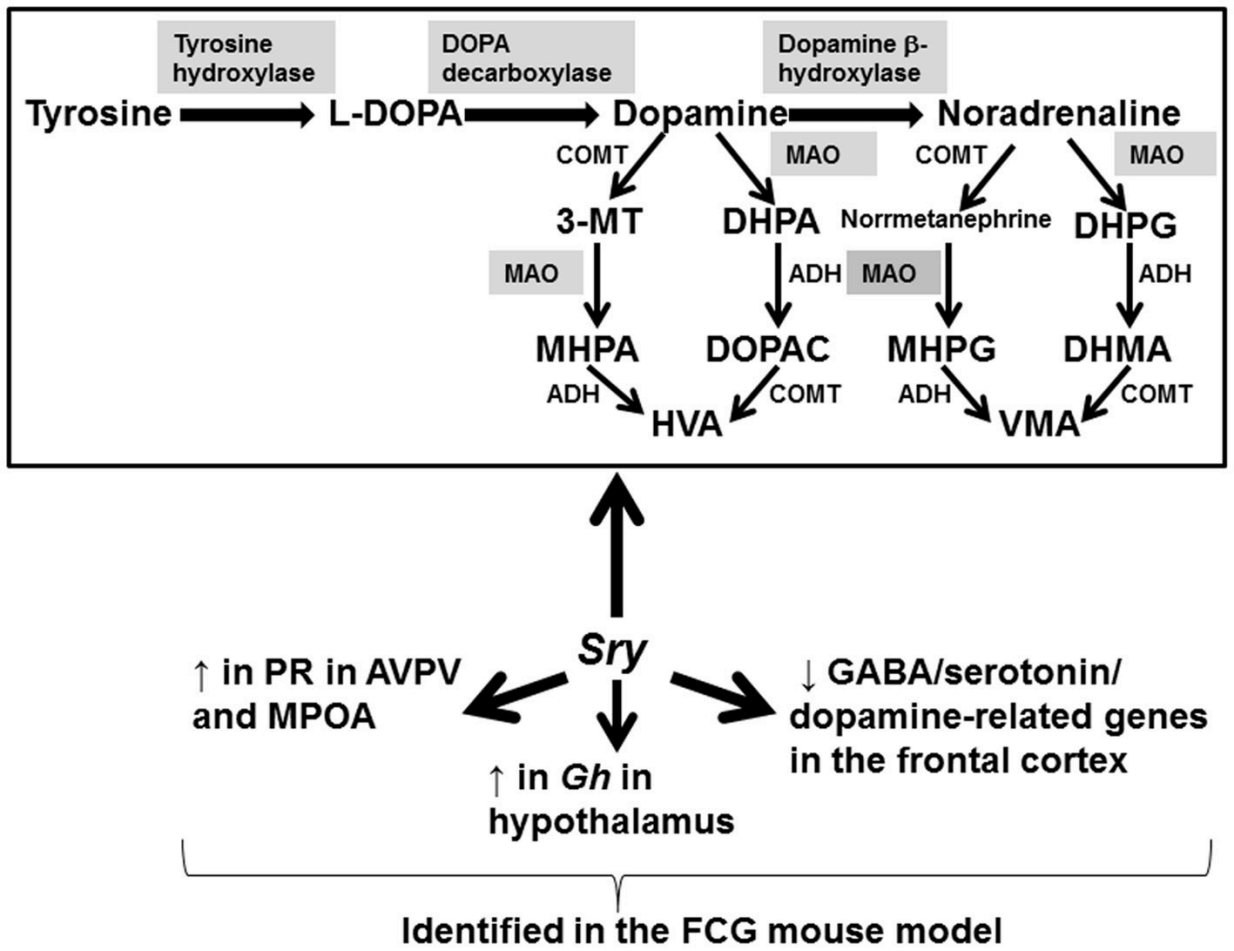
Genes, proteins, and pathways affected by SRY. Studies examining wild-type male mice, the FCG mouse model, and human brain samples/neuronal cells reveal these genes and pathways are primarily affected by the expression of *Sry*/*SRY*, respectively. Shaded enzymes are altered by *Sry*/*SRY* expression. 3-MT, 3-methoxytyramine; DHPA, 3,4 dihydrophenylacetaldehyde; MHPA, 3-methoxy-4-hydroxyphenylacetaldehyde; HVA, homovanillic acid; DHPA, 3,4-dihydroxyphenylacetic acid; DOPAC, 3,4-dihydroxyphenylacetic acid; MHPG, 3-methoxy-4-hydroxyphenylglycol; DHPG, dihydroxyphenylglycol; DHMA, 3,4-dihydroxymandelic acid; VMA, vanillylmandelic acid; ADH, alcohol dehydrogenase; MAO, monoamine oxidase; COMT, catechol-O-methyltransferase.

The focus of this review has been to examine the evidence to date that SRY is required for brain sexual differentiation. The evidence to date in laboratory rodents and humans suggest that Sry/*SRY* is expressed in certain brain regions in males, especially the cortex, midbrain, hypothalamus, and SN (Clepet et al., 1993; Lahr et al., 1995; Mayer et al., 1998, 2000; Dewing et al., 2006; Czech et al., 2012). The primary enzymes and pathways in the brain altered by SRY include TH, MAO A, and dopaminergic pathways (Milsted et al., 2004; Dewing et al., 2006; Wu et al., 2009; Czech et al., 2012, 2014; Figure 2). The latter effects of SRY might account for men being more susceptible for dopaminergic-based neurological disorders, e.g., Parkinson's disease and schizophrenia.

While SRY may be required for normal male brain function in many therian males, early induction of this gene can also impair neurogenesis as revealed by the hSRY^ON^ mice (Kido et al., 2017). The FCG mouse model has been useful in uncoupling the effects of *Sry* from other Y chromosome-associated genes (Wagner et al., 2004; Gatewood et al., 2006; Arnold and Chen, 2009; Chen et al., 2012; Kopsida et al., 2013; Kuljis et al., 2013; Seney et al., 2013; Quinnies et al., 2015). The collective results from this model indicate that *Sry* and other sex chromosome associated genes interact with steroid hormones to affect neurobehavioral programming as evidenced by the fact that the phenotypes observed depend upon whether the animals were intact or gonadectomized. For instance, gonadectomized XX females consumed more food and showed increased adiposity compared to XY mice (Chen et al., 2012). Conversely, intact XX and XY*Sry*^−^ mice demonstrated increased activity levels, consumed less food, and showed more anxiety-like behaviors (Kopsida et al., 2013). Social and parenting behaviors are also influenced by *Sry* and other Y chromosome associated genes (Gatewood et al., 2006; McPhie-Lalmansingh et al., 2008). The FCG mouse model reveals that *Sry* mediates neural expression of PR in the AVPV, MPOA, and ventromedial nucleus, GABA/serotonin/dopamine-related genes within the frontal cortex, and *Gh* expression in the hypothalamus (Figure 2; Wagner et al., 2004; Seney et al., 2013; Quinnies et al., 2015). Future transcriptomic and proteomic analyses of several brain regions within the FCG mouse model will likely elucidate other genes and pathways regulated by Sry. While this mouse model, along with the *Sry* KO mice generated via TALEN (Kato et al., 2013), have been useful in dissecting potential roles of *Sry*, future transgenic technology should specifically ablate *Sry* in brain neuronal cells. Such a deletion will allow further elucidation of the role of *Sry* in brain sexual differentiation and presumably distinguish secondary effects due to alterations in gonadal steroid hormones.

Surprisingly, there are rodent species within *Ellobius* and *Tokudaia* that have lost the Y chromosome and *Sry*. Such species might be useful in understanding how brain sexual differentiation can occur in the absence of *Sry*. While it is still not fully clear what presumably autosomally-associated genes might direct gonadal and brain sexual differentiation, several genes have been eliminated as possible candidates in both genus. These include *Zfy, Nrd5a1, Dax1, Df1, Foxl2/Pistr1, Sox9, Sox3, Dmrt1, Tspy, Eif2s3y*, and *Kdm5d* (Baumstark et al., 2001, 2005; Arakawa et al., 2002; Just et al., 2002, 2007; Kuroiwa et al., 2010). Two genes that might stimulate male gonad sexual differentiation in Amami spiny rats are *Cbx2* and *Er71* (Kuroiwa et al., 2011; Otake and Kuroiwa, 2016). However, it is not clear if these genes or possibly other transcripts are involved in brain sexual differentiation in this species and *Ellobius spp*. that lack *Sry*. To address this critical gap in our understanding, global transcriptomic profiles of the brain for these genuses and species would be useful. It would be of interest to determine whether similar transcripts are involved in programming the brain for both genuses and species devoid of the Y chromosome. On the other hand, it might be that brain sexual differentiation in the varying genuses and species is directed by unique signature pattern of transcript(s). Such findings would suggest that multiple pathways exist to sculpt brain masculinization even within therian mammals.

The prototherian mammals or monotremes further demonstrate that the presence of *Sry* is not a requisite for gonad and brain sexual differentiation as these species lack this gene. Both male and female duck-billed platypuses and echidnas contain five sets of X and Y chromosomes that resemble avian sex chromosomes (Rens et al., 2007; Wallis et al., 2008). As above, more genes have been discarded in searching for those that may regulate sexual differentiation in monotremes. The rejected gene pool includes *Sox3, Sox9, Sox10, Wt*1, *Sf1, Lhx1, Lhx2, Fgf9, Wnt4, Rspo1*, and *Gata4* (Grafodatskaya et al., 2007; Wallis et al., 2007a,b). *Amh* and *Crspy* remain as viable contenders in duck-billed platypuses, and possibly echidnas (Tsend-Ayush et al., 2012; Cortez et al., 2014). CRISPR/Cas9 deletion of *Amh* and/or *Crspy* might reveal one or both of these genes to be essential in initiating male sexual differentiation in monotremes.

While sexual differentiation in the above species is mediated through genetic (albeit not necessarily involving *Sry* or Y chromosome associated genes) sex determination, fish, and reptilian species can demonstrate GSD and/or TSD. Unlike most other taxa, sexualization in fish is exceptionally plastic, which may confer an evolutionary advantage in rapidly changing environmental conditions (Todd et al., 2016). The sex determining genes in fish appear to vary across species, and several *sox* forms have been implicated in governing sexual differentiation in diverse fish species (Ito et al., 1995; Takamatsu et al., 1995; Yokoi et al., 2002; Zhou et al., 2002; Wang et al., 2003; Navarro-Martin et al., 2009, 2012; Zhang et al., 2010; Uy et al., 2012; Reyhanian Caspillo et al., 2014; Jiang et al., 2015). Other gene networks regulating sexual differentiation have been well characterized in medaka. In males of this species, evidence suggests that *wt1a* upregulates *dmy*, which increases *gsdf* and *sox9a2* but suppresses the expression of *rspo1* (Nanda et al., 2002; Matsuda, 2003; Kikuchi and Hamaguchi, 2013; Chakraborty et al., 2016). The genes *dmrt1bY* and *dmrt1* also seemingly compensate for the absence of therian *Sry*. Other example genes that might regulate sexual differentiation of the gonad and brain in other fish species include *amhy, amhr2, sdY, wnt4b, cyp19a1a*, and *foxl2* (Alam et al., 2008; Guiguen et al., 2010; Le Page et al., 2010; Hattori et al., 2012; Kamiya et al., 2012; Myosho et al., 2012; Yano et al., 2012; Heule et al., 2014; Cui et al., 2017).

In those reptilian species that possess TSD, such American alligators, painted turtles and sea turtles, *wnt11, cebpa, trpv4, sf1, wt1, dax1, dmrt1*, and *sox9* may contribute to male gonad sexual differentiation (Torres Maldonado et al., 2002; Valenzuela et al., 2013; Yatsu et al., 2015, 2016). However, the same genes might not regulate gonad sexual differentiation across all those with TSD. While *dax1* might be involved in painted turtles, it is not a regulatory factor in sea turtles (Torres Maldonado et al., 2002; Valenzuela et al., 2013). The gene *sox9* might be important in sea turtles, but it is presumably not essential in alligators (Western et al., 1999a,b; Torres Maldonado et al., 2002). It is also not clear though if the same genes regulate brain sexual differentiation across these reptilian species. Female painted turtles derived from early exposure to BPA have increased expression of mitochondrial and ribosomal associated genes (Manshack et al., 2017).

While Australian central bearded dragons possess sex chromosomes, GSD can be overridden when eggs are incubated at higher temperatures (Deveson et al., 2017). Females derived from TSD but not those due to GSD have intron retention within *jarid2* and *jmid*, and the same introns are present in the embryonic gonads of TSD alligators and turtles. As introns are not transcribed, this inclusion of introns within these genes might induce sex-dependent epigenetic changes.

This latter study introduces the concept that *Sry* or other genes regulating brain sexual differentiation may operate via epigenetic changes. Such epigenetic modifications could entail those involving DNA methylation, histone protein modifications, or alteration in miRNAs. Rodent, zebrafish, European sea bass, and *Drosophila* studies have shown sex-dependent differences in all three types of epigenetic changes (Navarro-Martin et al., 2011; Jasarevic et al., 2012; Sato and Yamamoto, 2014; Shen et al., 2015; Bramble et al., 2016; Chatterjee et al., 2016; Forger, 2016; Kigar et al., 2016; Pfau et al., 2016; Morgan and Bale, 2017; Mosley et al., 2017). *Sry* may induce several epigenetic changes within the brain (Sekido, 2014). One example as discussed previously is that *circSry* might act as a “miRNA sponge” by binding to ncRNAs and thereby rendering them inactive, as might occur for miR-148 in brain neuronal cells (Obernosterer et al., 2006; Morton et al., 2008; Eskildsen et al., 2011; Hansen et al., 2013). To address the potential involvement of *Sry* in altering the brain epigenome, the DNA methylome, histone protein modifications, and miRNA profile should be screened in several brain regions of the FCG mouse model and future models lacking *Sry* in discrete brain regions or neuronal cell populations. Such approaches might also be considered in the *Tokudaia* and *Ellobius* species lacking *Sry*, monotremes, and fish/reptiles with TSD. By examining several taxa that include those with *Sry* and those without *Sry*, common epigenetic changes directing sexual differentiation across taxa might be revealed. In other words, while the emphasis to date has been on identifying genes regulating gonad and brain sexual differentiation, changes within DNA methylation, histone proteins, or miRNAs might instead be the common factor(s) driving gonad and brain sexual differentiation across vertebrate species. Future studies should be directed at using transgenic mice and naturally occurring animal models lacking *Sry* to address this exciting possibility.

The question posed at the outset was whether SRY is required for brain sexual differentiation. While there is evidence *SRY*/*Sry* is expressed and might affect certain pathways in the brain of men and select rodent models, respectively, it is also clear that brain sexual differentiation in therian and prototherian mammals, fish, reptiles, and likely in other vertebrate species occurs in the absence of this gene. However, it is uncertain which genes that presumably reside on autosomal chromosomes underpin brain sexual differentiation in these animals. Sry has been postulated to induce epigenetic changes in the brain, but it remains to be determined which ones are critical in modulating masculinization. Even less is known about the potential sex-dependent epigenetic cascade of events within the brain of species lacking *Sry* that might orchestrate masculinization or feminization of the brain. Such sexually dimorphic epigenetic modifications might be universal across taxa and possibly even precede the actions of *Sry*. In sum, a comparative taxa approach that examines atypical animal models where males are devoid of *Sry* might hold the key to unlocking other genes, pathways, and epigenetic changes essential in sculpting brain sexual differentiation.

## Author contributions

The author confirms being the sole contributor of this work and has approved it for publication.

### Conflict of interest statement

The author declares that the research was conducted in the absence of any commercial or financial relationships that could be construed as a potential conflict of interest.
